# Simultaneous Determination of Glyphosate, Aminomethylphosphonic Acid, and Glufosinate in Green Coffee Beans by LC-MS/MS: Optimization, Validation, and Field Study

**DOI:** 10.1021/acs.jafc.5c09979

**Published:** 2026-01-20

**Authors:** Júlio César R. M. da Silva, Millena Christie F. Avelar, Márcia C. M. Ribeiro, Mariana de O. Almeida, Vanessa H. F. de Faria, Vanessa M. Osório, Adriana F. Faria

**Affiliations:** † Department of Chemistry, Institute of Exact Sciences, 28114 Universidade Federal de Minas Gerais, 31270-901 Belo Horizonte, MG, Brazil; ‡ Pesticides Residues Laboratory − Fundação Ezequiel Dias, 30510-010 Belo Horizonte, MG, Brazil; § Department of Chemistry and Physics − Center of Exact, Natural and Health Sciences, 680695 Universidade Federal do Espírito Santo, 29500-000 Alegre, ES, Brazil

**Keywords:** polar herbicides, green coffee, QuPPe-PO, dispersive solid-phase extraction, validation

## Abstract

Despite the widespread use of glyphosate in coffee crops, there is a lack of simple, low-cost, validated methods for detecting glyphosate and related compounds in green coffee beans, the primary export form subject to international regulation. This study optimized and validated a liquid chromatography with tandem mass spectrometry method for the determination of glyphosate, aminomethylphosphonic acid (AMPA), and glufosinate in green coffee, using a modified QuPPe-PO extraction followed by a cleanup combining liquid–liquid extraction and dispersive solid-phase extraction. Validation followed SANTE/11312/2021 guidelines, confirming the method’s linearity, precision, accuracy, and quantification limits. Limits of quantification were 0.02 mg kg^–1^ (glyphosate), 0.04 mg kg^–1^ (AMPA), and 0.01 mg kg^–1^ (glufosinate). The method was applied to a field study with Arabica coffee and Conilon Coffee, revealing that plant height was the primary factor influencing residue levels. The proposed method enables reliable monitoring of polar herbicides in coffee without the need for derivatization.

## Graphical abstract

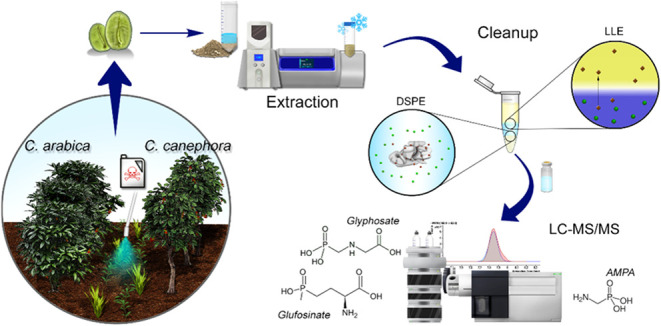

## 1 Introduction

Coffee is one of the most widely consumed beverages worldwide and, in recent decades, it has evolved from being a simple commodity to a specialty product, with a direct impact on its production and commercialization.^1^ This is due to the fact that quality of the beverage is intrinsically related to the quality of the beans.^2^ Brazil is the main producer and exporter of coffee, with Arabica coffee (*Coffea arabica*) accounting for 74.9% of exports, followed by Conilon coffee (*Coffea canephora*) at 17.4%.^3,4^

Coffee plants are sensitive to competition from weeds for water, light, and nutrients, which compromises their growth and productivity.^5^ As a result, the use of herbicides is widespread in coffee crops to control these competitors. However, pesticides spraying may lead to residue contamination of food, either through direct deposition or via spray drift. In addition, it can accumulate in the soil and be absorbed by plants.^6^ Furthermore, recent studies revealed that exposure to glyphosate can alter metabolism and lead to intestinal and systemic diseases, such as Crohn’s and Alzheimer’s disease.^7^

Glyphosate (C_3_H_8_NO_5_P) is a postemergent, systemic, nonselective, broad-spectrum, organophosphorus compound, and it is the most widely used herbicide globally.^8^ In the environment, glyphosate undergoes chemical, physical, or biological degradation processes, with aminomethylphosphonic acid (AMPA – CH_6_NO_3_P) being its main metabolite.^9^ Another herbicide with similar characteristics to glyphosate, though less widely used, is glufosinate (C_5_H_12_NO_4_P). It is an organophosphorus, postemergent, contact, nonselective, broad-spectrum herbicide.^10^

Glyphosate and glufosinate are classified as highly polar pesticides (HPPs), which is challenging for quantitative analysis.^11^ Their high polarity makes them incompatible with traditional multiresidue extraction methods, which typically rely on acetonitrile as the extraction solvent.^12,13^ Furthermore, the high polarity of these analytes results in poor retention on conventional reversed-phase chromatographic columns.^12^

In addition to the challenges posed by HPP analytes, the coffee matrix itself presents analytical challenges due to its endogenous components. Coffee is classified as a difficult or unique commodity owing to its complex composition.^14,15^

In the literature, there are several published works on the determination of pesticide residues in green coffee beans or soluble coffee; however, these do not include HPPs.^16–21^ Recently, the gap in the determination of glyphosate in coffee has begun to be addressed.^22–25^ A study on the degradation of glyphosate during the coffee roasting process was carried out by Delatour et al.^22^ Similarly, the effects of roasting and beverage preparation on glyphosate and AMPA content were evaluated by Bytof et al.^23^ The presence of glyphosate and AMPA residues in coffee leaves was investigated by Schrübbers et al.^24^ An analytical method for determining glyphosate in green coffee was proposed by Paiva et al.^25^ The four cited studies performed analyses using liquid chromatography with tandem mass spectrometry (LC-MS/MS). The first three employed derivatization to enhance retention of glyphosate and/or AMPA on the C18 columns, while the last study used a Restek Raptor Polar column. This work sought to develop a simpler and faster method, without a derivatization step and with sample preparation using smaller volumes of less toxic solvents. Therefore, a LC-MS/MS method using hydrophilic interaction liquid chromatography (HILIC) column was optimized and validated for the simultaneous determination of glyphosate, AMPA and glufosinate in green coffee beans. The limit of quantification met the maximum residue limits (MRLs) established by different regulatory agencies: the National Health Surveillance Agency of Brazil (1.0 mg kg^–1^ for glyphosate and 0.5 mg kg^–1^ for glufosinate),^26^ the Environmental Protection Agency of the USA (1.0 mg kg^–1^ for glyphosate)^27^ and the European Food Safety Authority of the European Union (0.1 mg kg^–1^ for glyphosate and 0.1 mg kg^–1^ for glufosinate).^28^ Moreover, the method was applied to the analysis of samples from a field study, in which glyphosate was applied to crops with different spray nozzles, number, and frequency of application.

## 2 Materials and Methods

### 2.1 Reagents and Materials

Ultrapure water, 18.2 MΩ cm, was purified by a Milli-Q system (Millipore, Massachusetts, USA). Acetonitrile (ACN) (99.8%) and methanol (MeOH) (99.9%), HPLC grade, were acquired from J. T. Baker (New Jersey, USA). Formic acid (98–100%) and ethyl acetate (EtOAc) (99.8%) were purchased from Merck (Darmstadt, Germany). Methyl *tert*-butyl ether (MTBE) (99.8%), HPLC grade, was obtained from Honeywell (North Carolina, USA). Ethylenediaminetetraacetic acid dihydrate (EDTA) (P. A.) was purchased from Química Moderna (São Paulo, Brazil). InfinityLab Deactivator additive was acquired from Agilent (California, USA). Hexane (96.0%), HPLC grade, was obtained from Scharlau (Barcelona, Spain). C18 cartridges (500 mg, 6 cm^3^) were purchased from Applied Separation (Pennsylvania, USA). Oasis HLB (hydrophilic–lipophilic balance) cartridges (200 mg, 6 cm^3^) were obtained from Waters (Massachusetts, USA).

### 2.2 Standards

Stock solutions of the standards were prepared in MeOH at a concentration of 5.0 μg mL^–1^ for glufosinate (Dr. Ehrenstorfer, Augsburg, Germany), 10.0 μg mL^–1^ for glyphosate (Restek, Pennsylvania, USA) and AMPA (Dr. Ehrenstorfer, Augsburg, Germany). Working solutions were prepared by mixing the stock solutions and diluting them 10-fold in MeOH/H_2_O (1:1 v/v). All standards were prepared in polypropylene flasks to minimize potential analyte losses due to adsorption. Polypropylene conical centrifuge tubes, microtubes, and vials were also used throughout sample preparation and analysis to prevent adsorption of glyphosate, AMPA, and glufosinate onto container surfaces.

### 2.3 Coffee Samples

Control coffee samples of the species Arabica coffee and Conilon coffee were obtained from crops in which no pesticides were used. A field study was conducted by the Capixaba Institute for Research, Technical Assistance and Rural Extension, in which the herbicide RoundUp was used in seven treatments for species Arabica coffee and Conilon coffee, varying the number and months of application and type of nozzle used for spraying (Table 1). All treatments were applied in three blocks (replicates) with 8 coffee plants each. The coffees were harvested in March of 2024. Green coffee beans from this field study were analyzed using the method developed in this work.

**Table 1:** Glyphosate Treatments Applied on Arabica Coffee and Conilon Coffee Crops^a^

Treatment	Nozzle	Number of applications	Months
TR1	FM	2	October and December (2023)
TR2	FM	2	October (2023) and February (2024)
TR3	FM	3	October, December (2023) and February (2024)
TR4	SFF	2	October and December (2023)
TR5	SFF	2	October (2023) and February (2024)
TR6	SFF	3	October, December (2023) and February (2024)
TR7	Not Applied (control sample)		

a TR: treatment; SFF: standard flat fan; FM: foam marker.

### 2.4 Instrumental Conditions

LC-MS/MS analyses were executed on the Agilent Series 1200 SL series liquid chromatography system equipped with an autosampler and a quaternary pump liquid chromatograph coupled to an Agilent 6495A triple quadrupole mass spectrometer equipped with an electrospray ionization (ESI) featuring iFunnel and Agilent Jet Stream technologies (Agilent, California, USA). Instrument control and data treatment were performed by Agilent Masshunter Worksation Data Acquisition and Quantitative Analysis softwares.

Analytes separation was performed by an Anionic Polar Pesticide (APP) column (2.1 × 100 mm, 5 μm and 130 Å) (Waters, Massachusetts, USA), operated at 50 °C and with an injection volume of 10 μL. The mobile phases were ultrapure water with 0.9% (v/v) of formic acid and 0.1% (v/v) of InfinityLab Deactivator additive (Phase A) and ACN with 0.9% (v/v) of formic acid (Phase B). The flow rate was fixed at 0.5 mL min^–1^, and the gradient was as follows: 0.00–3.50, 5% A; 3.51–5.00, 5–95% A; 5.01–12.00, 95% A; 12.01–12.50, 95–5% A; 12.50–16.00, 5% A.

Detection was performed using multiple reaction monitoring (MRM), and an ESI interface operating in both positive and negative modes. Spectrometric and source parameters are shown in Table 2. Fragmentation conditions were optimized by direct injection of 0.5 μg mL^–1^ of glufosinate and 1.0 μg mL^–1^ of AMPA and glyphosate in MeOH/H_2_O (1:1 v/v) at a flow rate of 0.5 mL min^–1^. The parameters were automatically determined by the Agilent MassHunter Optimizer software.

**Table 2:** Optimized Spectrometric, Source, and Acquisition Conditions for Analytes^a^

Spectrometric and source parameters	
Model	Agilent 6495 Triple Quadrupole
Ionization mode	AJS/ESI
Gas temperature	120 °C
Gas flow	15 L min^–1^
Nebulizer pressure	39 psi
Sheath gas temperature	375 °C
Sheath gas flow	12 L min^–1^
Capillary voltage	(+) 3500 V; (−) 3000 V
Nozzle voltage	(+) 300 V; (−) 500 V
High Pressure RF	(+) 200 V; (−) 105 V
Low Pressure RF	(+) 70 V; (−) 60 V
Electron multiplier voltage (EMV)	(+) 780 V; (−) 1800 V

Analyte	Ionization mode	RT (min)	Precursor ions (*m/z*)	Product ions (*m/z*)	CE (V)
AMPA	ESI–	6.07	110	63^q^	20
			110	81^c^	8
Glyphosate	ESI–	7.98	168	63^q^	36
			168	81^c^	16
Glufosinate	ESI+	6.19	182	119^q^	16
			182	136^c^	12

a ^q^ quantification transition. ^c^ confirmation transition; RT: retention time; CE: collision energy.

### 2.5 Instrumental Condition Optimization

During the chromatographic condition optimization, the following were evaluated: (1) stationary phase XSelect CSH Fluoro-Phenyl (2.1 mm × 100 mm, 5 μm) (Waters, Massachusetts, USA) and APP; (2) mobile phase composition; (3) elution gradient.

The spectrometric and ionization source parameters were optimized by Plackett-Burman design using ± 30% range from the initial parameters (Table 3). The multiple response vector was used as the response for design and was estimated by the sum of the ratios of the area of each analyte in the test by the highest area obtained for this analyte in all tests performed (Table S1).

**Table 3:** Experimental Variables Studied at Low (−1) and Upper (+1) Levels in the Placket-Burman Design^a^

	Level			
Factors	–1	+1	Contrasts	Confidence interval (α = 0.05)
Delta EMV (+) (V)	420	780	0.503	±0.246
Delta EMV (−) (V)	1050	1800	1.051	±0.246
Gas temperature (°C)	100	156	–0.023	±0.246
Gas flow (L min^–1^)	10.5	19.5	0.079	±0.246
Pressure (psi)	21	39	0.317	±0.246
Sheath gas temperature (°C)	262.5	400	0.075	±0.246
Sheath gas flow (L min^–1^)	8.4	12	–0.061	±0.246
Capillary (+) (V)	2450	4550	–0.074	±0.246
Capillary (−) (V)	2100	3900	0.020	±0.246
Nozzle (+) (V)	300	390	–0,017	±0.246
Nozzle (−) (V)	350	650	0.272	±0.246
Low RF (+) (V)	70	130	–0.306	±0.246
Low RF (−) (V)	42	78	0.076	±0.246
High RF (+) (V)	105	195	–0.032	±0.246
High RF (−) (V)	105	195	–0.275	±0.246

a EMV: Electron multiplier voltage; RF: Radiofrequency; High RF: High pressure chamber RF; Low RF: Low pressure chamber RF; Nozzle: voltage applied to the nozzle’s nebulizer; (±): ESI ionization mode; standard deviation = 0.128 e *t*_(0.05; 4)_ = 2.776.

### 2.6 Sample Preparation

Green coffee beans were ground in a Retsch Ultra Centrifugal Mill ZM 300 (Düsseldorf, Germany) with a 1.0 mm sieve. Then, 2.000 g of the processed sample was transferred to a 50 mL polypropylene centrifuge tube, to which 10.0 mL of water and 10.0 mL of MeOH, both acidified with 1.0% (v/v) formic acid, were added. The mixture was mechanically stirred in a Geno/Grinder 2010 (SPEX SamplePrep, New Jersey, USA) at 1,700 rpm for 5 min, then stored at −80 °C for 30 min (freeze-out). Next, the extract was centrifuged in a ThermoFisher Scientific Multifuge X4R Pro (Dreieich, Germany) at 4,200 rpm at −10 °C for 10 min. For cleanup, 1.0 mL of the supernatant was transferred to a microtube containing 75.00 mg of C18 and 750 μL of MTBE/EtOAc (9:1 v/v). The mixture was vortexed for 30 s to resuspend the C18 particles, then mechanically shaken at 1300 rpm for 2.5 min and then centrifuged at 13,000 rpm at 0 °C for 5 min. Finally, 500 μL of the aqueous phase was collected, filtered through a 0.22 μm PVDF membrane, diluted 2.5-fold in ACN/H_2_O (8:2 v/v) and injected (10 μL) into the LC-MS/MS system.

### 2.7 Sample Preparation Optimization

The first evaluation performed was the addition of 1.0 mL of 10% m/v EDTA solution to samples spiked with glyphosate at 1.0 mg kg^–1^, as recommended by the Quick Polar Pesticides method for Food of Plant Origin (QuPPe-PO).^29^ The study was conducted in six replicates using Arabica and Conilon coffee to investigate the need for adding EDTA solution before extraction, considering its potential role in preventing metal complexation (e.g., with calcium or magnesium) that could affect the analytical response of glyphosate. A second study was performed to evaluate the cleanup of extracts by means of liquid–liquid extraction (LLE), solid-phase extraction (SPE), and dispersive solid-phase extraction (DSPE). A simplex-centroid mixture design was applied to determine the optimal extraction solution composition for LLE by evaluating hexane, EtOAc, and MTBE. Seven tests were performed, each using a final volume of 750 μL, where the vertices were the pure solvents, in addition to intermediate points 1:1 and the centroid 1:1:1. SPE and DSPE were evaluated alone and in combination with LLE (Figure S1). The SPE investigation in order to retain matrix interferents was performed using HLB (200 mg, 6 cm^3^) and C18 (500 mg, 6 cm^3^) cartridges. The cartridges were preconditioned and then 1.0 mL of the extract spiked with 1.0 μg mL^–1^ was eluted and collected in microtubes. For DSPE evaluation, 75.00 mg of C18 and 1.0 mL of the sample were added to a microtube. The mixture was mechanically shaken at 1100 rpm for 2.5 min, centrifuged at 13,000 rpm at 0 °C for 5 min, and the supernatant was collected. All extracts were filtered through a 0.22 μm PVDF membrane and injected (10 μL) into the LC-MS/MS system.

### 2.8 Method Validation

The method validation was performed in accordance with the EU SANTE/11312/2021.^14,15^ Matrix effect was estimated through the ratio between the slopes of the matrix-matched and external calibration curves. Variations within ± 20% were considered an indicative of no significant matrix effect. To evaluate linearity, matrix-matched calibration curves were prepared at six concentration levels by adding the working solutions to the extract from a mixture of Arabica and Conilon coffee (1:1 w/w). The linear range varied from 0.01 to 0.07 mg kg^–1^ for glufosinate, 0.02 to 0.14 mg kg^–1^ for glyphosate and from 0.04 to 0.14 mg kg^–1^ for AMPA. The presence of outliers in the data set was assessed by the Jack-Knife standardized residuals test. Residual normality was verified by the Ryan-Joiner test, and variance homogeneity was evaluated using the Brown-Forsythe test. Analysis of variance (ANOVA) was used to verify the regression significance and the lack of fit of the model. Trueness and precision were assessed by estimating the recovery and the relative standard deviation (RSD) at a low, medium and high concentration levels, each in sextuplicate. This procedure was performed over three independent validation days, in which the combined data set corresponds to the intermediate precision condition. The limit of quantification (LOQ) was defined as the lowest concentration on the calibration curve that met the precision (RSD < 20%) and recovery (70–120%) criteria. LOD was calculated as 3.3 times the standard deviation of the blanks divided by the slope of the calibration curve.^30^

## 3 Results and Discussion

### 3.1 Instrumental Condition Optimization

The chromatographic separation of glufosinate, glyphosate, and AMPA is challenging due to the high polarity of these analytes,^13^ which results in low retention on C18 columns, the most commonly used stationary phase in liquid chromatography.^31^ Therefore, other columns were evaluated, such as XSelect CSH Fluoro-phenyl, which features a modified silica stationary phase bonded to a fluoro-phenyl group. The electronegativity of fluorine confers high polarity to the C–F bonds, enhancing retention of acidic compounds through dipole–dipole interactions and hydrogen bonds.^32^ In this column, chromatographic separation of the three analytes in solvent was achieved; however, the presence of matrix caused significant signal suppression and chromatographic peak deformation (Figure S2).

Another column evaluated was APP, which features a stationary phase with diethylamine functional group bonded to a hybrid silica particle. This chemical modifier allows separation by HILIC and weak anion exchange modes.^33^ The column is designed to effectively retain and separate polar anionic compounds, such as the analytes under study. The gradient was optimized and resulted in good retention and separation of glufosinate, AMPA and glyphosate. However, peak symmetry remained poor, with split peaks and tailing still observed. In an attempt to improve the peak shape, 5.0 μmol L^–1^ of the InfinityLab Deactivator additive was added to the aqueous mobile phase. The analytes contain phosphonic groups that can interact with the metal sites of the chromatographic system.^34,35^ Mendronic acid present in this additive can deactivate these sites, thereby inhibiting interaction with the analytes. The results showed an increase in the analytes retention time of the analytes and a significant improvement in peak shape (Figure 1). Thus, the composition of the mobile phase was defined as ultrapure water with the addition of 0.9% (v/v) formic acid and 0.1% (v/v) InfinityLab Deactivator additive and ACN acidified with 0.9% (v/v) formic acid.

**Figure 1:**
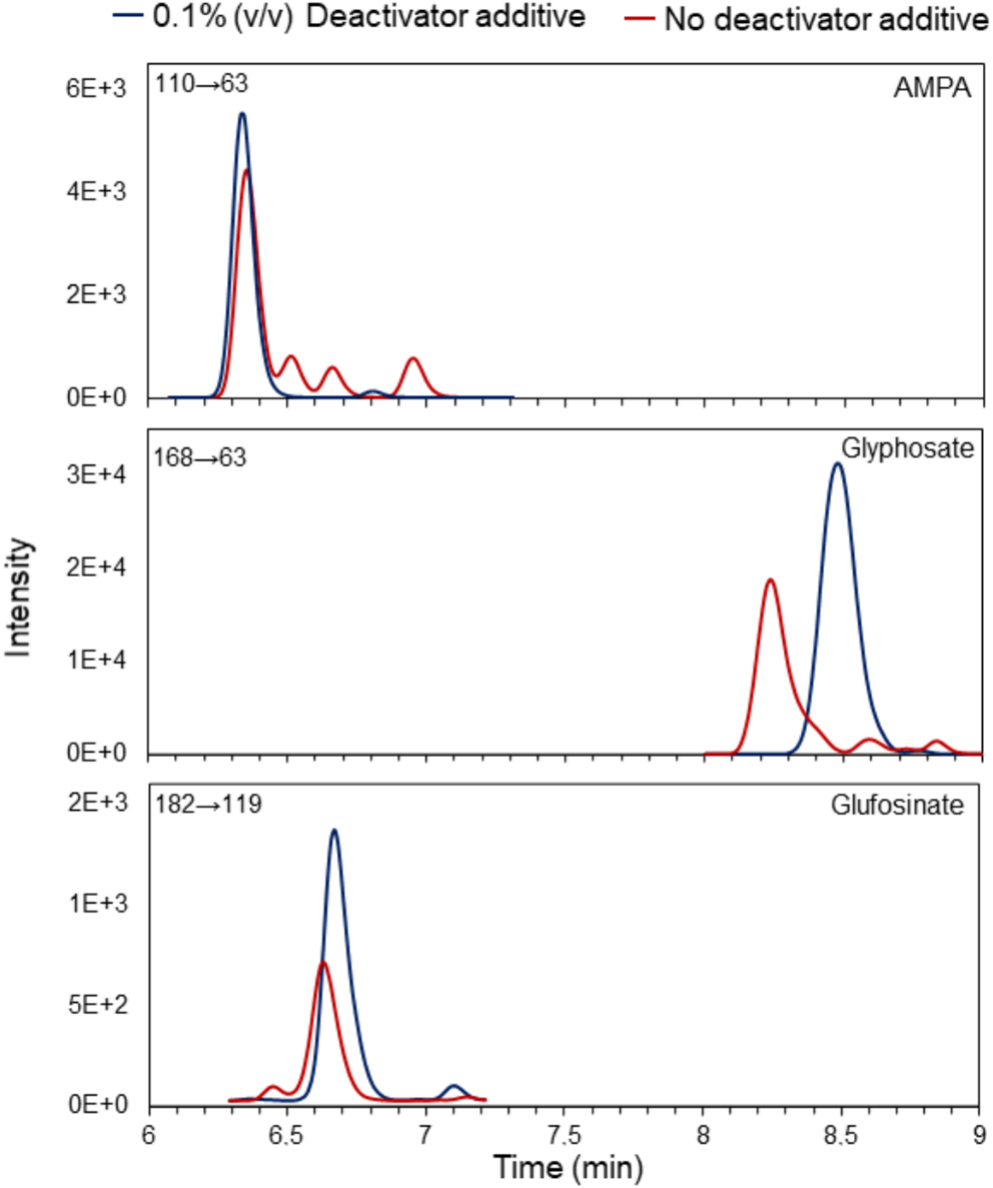
Chromatograms comparing the effect of the InfinityLab Deactivator additive in the aqueous mobile phase on the analyte signals: AMPA, glyphosate (1.0 μg mL^–1^) and glufosinate (0.5 μg mL^–1^).

In the optimization of the spectrometric conditions through the Plackett-Burman design, the contrasts were estimated using the multiple response vector as the response. The confidence intervals for contrasts were estimated and six factors (EMV (+), EMV (−), Pressure, Nozzle voltage (−), High RF (−) and Low RF (+)) were found to be significant at the 95% confidence level (Table 3). Thus, these parameters were changed as indicated by the design, while the others were maintained at the initial conditions. The results showed a significant increase in the analytical signal (Figure S3) and these parameters were defined for analyte acquisition (Table 2).

### 3.2 Sample Preparation Optimization

Sample preparation was based on the QuPPe-PO method,^29^ a procedure designed for the analysis of HPP in food. However, as the method does not include specific guidelines for the coffee matrix, optimization of the extraction and cleanup steps was necessary. The QuPPe-PO method recommends the use of a 10% (w/v) EDTA solution in the extraction step for cereals, pulses, nuts and oily seeds to eliminate possible interferences caused by metals,^29^ since glyphosate can form complexes with these elements.^36,37^ To ensure matrix-related relevance, both Arabica and Conilon coffee varieties were evaluated, as their distinct chemical compositions can influence metal complexation and matrix effects.^37,38^ Contrary to expectations, the results showed that the addition of EDTA solution in the extraction step reduced the glyphosate signal by 62 ± 8% and 59 ± 6% in Arabica coffee and Conilon coffee, respectively. This reduction is likely due to competition between EDTA and analytes for charges generated during the ESI ionization process, resulting in ionic suppression of the target compounds.^39^

HPPs such as glyphosate are soluble in aqueous solutions. Therefore, the use of LLE with organic solvents can be an alternative for extract cleanup. This approach was evaluated using solvents (hexane, EtOAc, and MTBE) with different log *K*_ow_ values, which form biphasic systems in aqueous medium and do not solubilize the analytes. The response surface (Figure 2) shows that the *x*_1_ *x*_2_ interaction region demonstrates an antagonistic effect between hexane and MTBE, while the *x*_1_ *x*_3_ and *x*_2_ *x*_3_ regions show the synergistic effect between hexane and EtOAc, as well as between EtOAc and MTBE. The design results indicated two conditions to maximize response: pure MTBE and MTBE/EtOAc (9:1 v/v). Evaluation of these two conditions showed that the use of the mixture resulted in an approximately 20% increase in the peak area of glyphosate. Additional experiments evaluated extractant volumes of 100, 250, 500, 750, and 1000 μL, with 750 μL providing the best performance and therefore being selected for the study.

**Figure 2:**
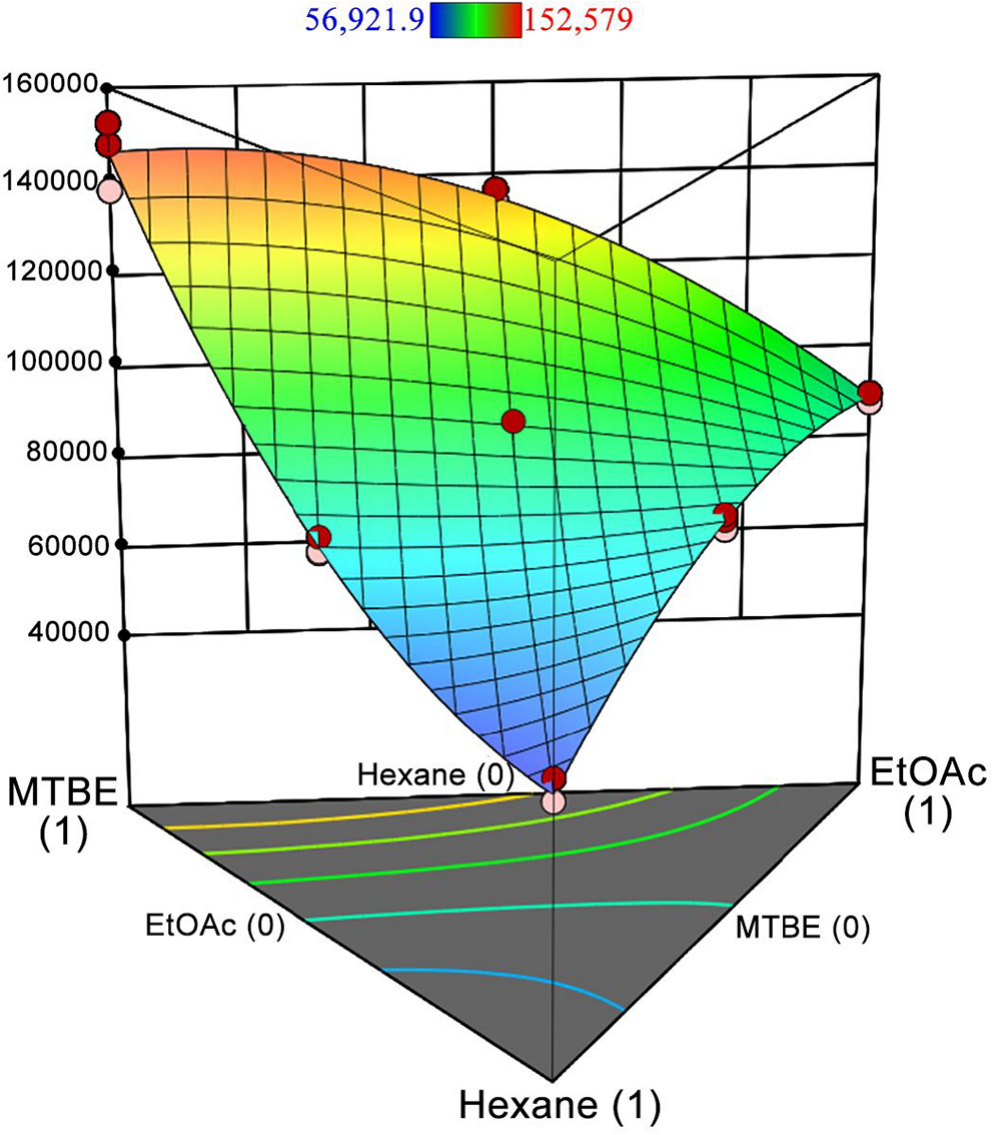
Response surface of the simplex–centroid mixture design for glyphosate (1.0 μg mL^–1^) treated with 750 μL of LLE solvents (*n* = 3). Main effects for the solvents are defined as *x*_1_ = hexane, *x*_2_ = EtOAc, *x*_3_ = MTBE.

In the literature, there are several studies that successfully employed SPE and DSPE to cleanup of plant extracts derived from matrices with low water content.^40–45^ Therefore, SPE using C18 and HLB cartridges and DPSE using C18 as sorbent were evaluated for the cleanup of coffee extract, as well as their combination with LLE, previously optimized. The results from this set of experiments showed that SPE using C18 cartridge (853,109 ± 49,409 au) and the combination of DSPE and LLE (828,628 ± 41,950 au) provided the greater chromatographic peak areas for glyphosate. Therefore, a *t* test was performed to compare the mean peak areas obtained under both conditions. The results showed no statistically significant difference at the 95% confidence level (*t*_calc_ = 0.654 < *t*_(0.025; 4)_ = 2.776). Thus, the DSPE combined with LLE was selected as a cleanup method for coffee, as it provided statistically equivalent results to SPE using a C18 cartridge, while offering practical advantages such as simpler execution, faster processing, and lower cost. A schematic of the optimized sample preparation procedure is shown in Figure 3.

**Figure 3:**
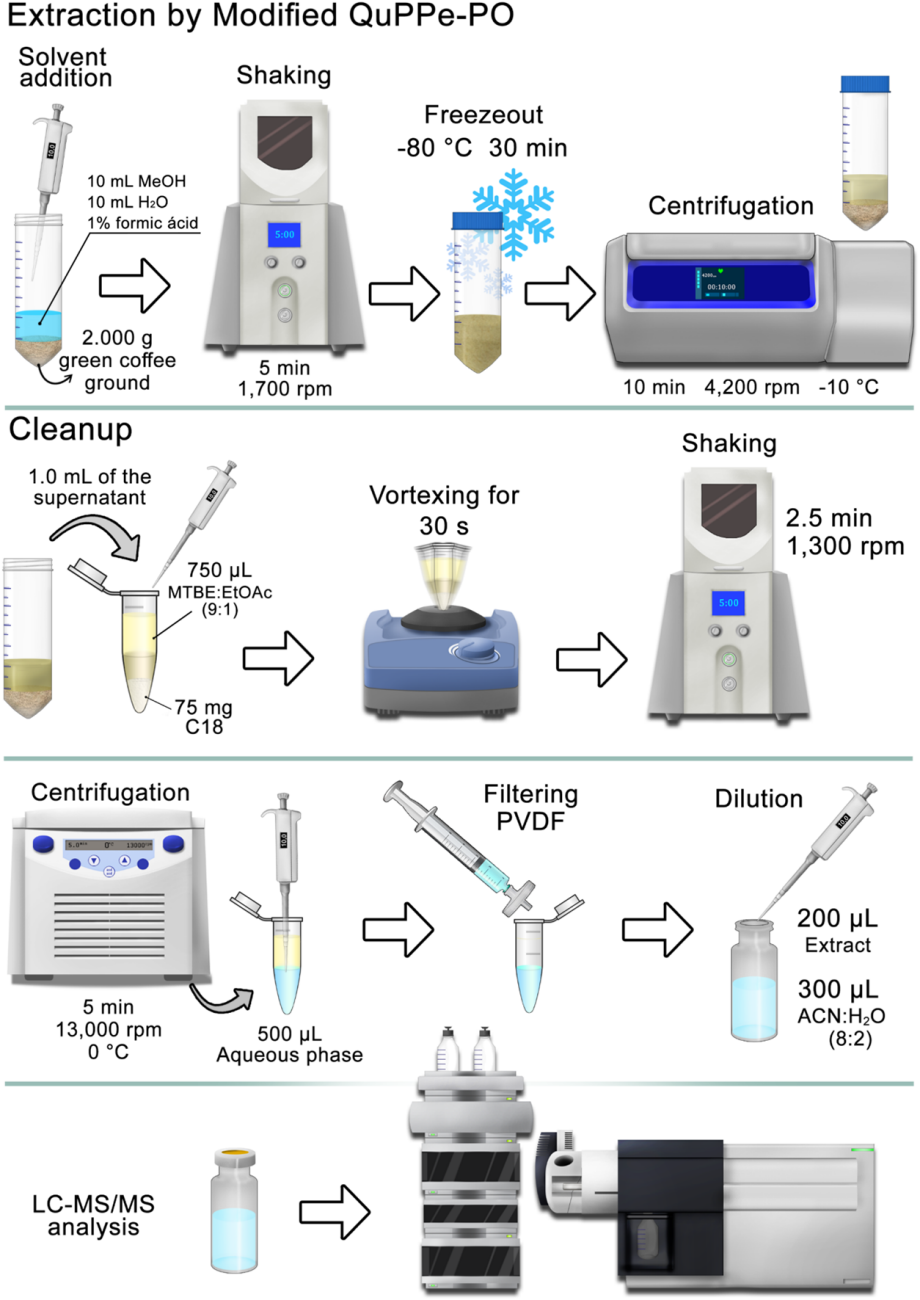
Flowchart of the optimized sample preparation process.

### 3.3 Validation

The matrix effect study showed a 71% suppression of the AMPA analytical signal, whereas glyphosate and glufosinate displayed a signal suppression of approximately 1%. Therefore, matrix-matched calibration was adopted to compensate for the matrix effect for AMPA determination. In the linearity assessment, the Ryan-Joiner test indicated that the deviations from normality were not significant, suggesting that the residuals for all three analytes followed a normal distribution. The Brown-Forsythe statistic showed the homoscedastic behavior of the variances, so the least-squares method was adopted for regression of the curves. ANOVA results showed that the regressions were statistically significant, with no evidence of lack of fit, therefore, confirming the adequacy of the linear model for the analytes studied. LOQ values complied with MLRs indicated by Regulation (EC) No. 396/2005,^28^ thus it is possible to ensure quantification within the recommended levels (Table 4). LOD values allowed reliable differentiation of analyte signal from baseline noise (Table 4). The results from precision and recovery (Table 4) met the established acceptance criteria, i.e., recoveries between 70–120% and RSDs below 20%. Therefore, the trueness and accuracy of the method were considered adequate.

**Table 4:** Method Validation Parameters Including Matrix Effect, Linearity, Limit of Quantification (LOQ), Recovery (*R*), and Intermediate Precision (RSD)^a^

Analyte	Matrix effect (%)	*R* ^2^	*F*_Reg_	*F*_LoF_	LOD (mg kg^–1^)	LOQ (mg kg^–1^)	Level (mg kg^–1^)	*R* (%)	RSD (%)
AMPA	71	0.9860	846.02	0.254	0.001	0.04	0.04	70	15
							0.10	77	6
							0.14	77	9
Glyphosate	1	0.9954	2589.68	1.914	0.003	0.02	0.02	77	8
							0.10	76	6
							0.14	77	7
Glufosinate	1	0.9748	618.89	0.374	0.003	0.01	0.01	87	9
							0.05	81	5
							0.07	83	8

a **F**_**Reg**_: *F*-value estimate for linear regression significance, while *F*_(0.05; 16; 1)_ = 4.747 is the critical *F*-value for AMPA and glyphosate and *F*_(0.05; 16; 1)_ = 4.494 for glufosinate; **F**_**LoF**_: *F*-value calculated to assess the lack of fit for the model, while *F*_(0.05; 8; 4)_ = 3.838 is the critical *F*-value for all three analytes.

There are few recent studies in the literature that describe the preparation and analysis of glyphosate and AMPA in coffee samples.^22–25^ However, none of them includes glufosinate. Bytof et al.^23^ proposed the determination of glyphosate and AMPA in green and roasted coffee by LC-MS/MS using a C18 column and derivatization of the analytes with 9-fluorenylmethoxycarbonyl chloride. The authors reported recoveries of 96.8%, intermediate precision in terms of RSD of 4.8% and LOQ of 0.01 mg kg^–1^ for glyphosate. However, the method presents high costs and low analytical throughput, due to need for derivatization, which demands prolonged incubation time (overnight). Additionally, employing hydrochloric acid requires a neutralization step with potassium hydroxide, plus a posterior cleanup with C8 cartridge, prior to derivatization step.

Delatour et al.^22^ did not provide detailed information on the chromatographic conditions to LC-MS/MS or the method used on the extraction step in their work. Although, the authors briefly mentioned a cleanup step involving dichloromethane and the use of 9-fluorenylmethyl chloroformate derivatization prior to analysis. They reported recoveries between 93 and 107%, intermediate precision with RSD below 11 and 18% for AMPA and glyphosate, respectively, and LOQ of 0.01 mg kg^–1^ for both analytes.

The method proposed by Paiva et al.^25^ for glyphosate analysis in green coffee beans achieved LOD and LOQ of 0.16 and 0.48 mg kg^–1^, respectively. Recoveries ranged from 92 to 112%, and intermediate precision (RSD) ranged from 5.74 to 9.05%. Although this method does not require a derivatization step, it involves the use of relatively large volumes of dichloromethane during sample preparation. While effective for removing coextracted matrix components, dichloromethane generates toxic and nonbiodegradable waste, which is inconsistent with the principles of green chemistry.^46^

In the method proposed by Schrübbers et al.^24^ for glyphosate analysis in coffee leaves treated with the herbicide, sample preparation involved the use of dichloromethane and hydrochloric acid. The cleanup of the extract was performed by SPE with Strata X cartridges, previously conditioned with 10 mL MeOH and 10 mL of 0.1% (v/v) formic acid. Then, the pH was adjusted to 9 for derivatization with 9-fluorenylmethyl chloroformate.

In this work, the validated method achieved satisfactory results for LOD and LOQ, complying with the MRLs for the analytes while using minimal volumes of less toxic solvents such as MeOH, MTBE, and EtOAc. Furthermore, the simple procedure, which does not require compound derivatization or SPE cartridges for cleanup, provided high analytical throughput and low operational cost, making it suitable for routine analysis of glyphosate, AMPA, and glufosinate in laboratory settings.

### 3.4 Field Study Sample Analysis

The analysis results for Conilon coffee samples from all seven treatments (Table 1) showed glyphosate signal responses below the established LOQ. In Arabica coffee, there were four treatments with levels above the LOQ (Figure 4). The higher glyphosate residue observed in Arabica coffee samples can be attributed primarily to plant height. As Arabica coffee plants are shorter than Conilon coffee ones, they are more susceptible to contamination by spray drift, given that the herbicide is applied close to the ground. Plants with shorter height tend to exhibit higher concentrations of glyphosate in their tissues, since this herbicide is mainly absorbed through the leaves,^47,48^ and the greater the leaf area close to the soil, the higher the likelihood of contamination by drift. Furthermore, once absorbed by the leaves, glyphosate can be translocated via the phloem to other plant organs, particularly those with higher metabolic activity, such as fruits.^49,50^ In addition to the height of the coffee tree, the type of nozzle and the interval between the last herbicide application and harvest also influenced glyphosate residue levels. Treatments 5 and 6 resulted in the highest levels of glyphosate concentrations. Both treatments used a standard flat fan nozzle and had a one-month interval between the last application and harvest. This type of nozzle produces wider spray pattern and smaller droplets, which favors the drift phenomenon.^51–53^ Variations in concentrations between replicates of the same treatment is likely due to drift, as manual application is subject to operational variables and wind conditions during application. The control samples of both species showed contamination levels close to LOD, likely due to the pesticide drift. AMPA and glufosinate were not detected, which is consistent with the composition of the commercial formulation used (Roundup), containing glyphosate as the sole active ingredient.

**Figure 4:**
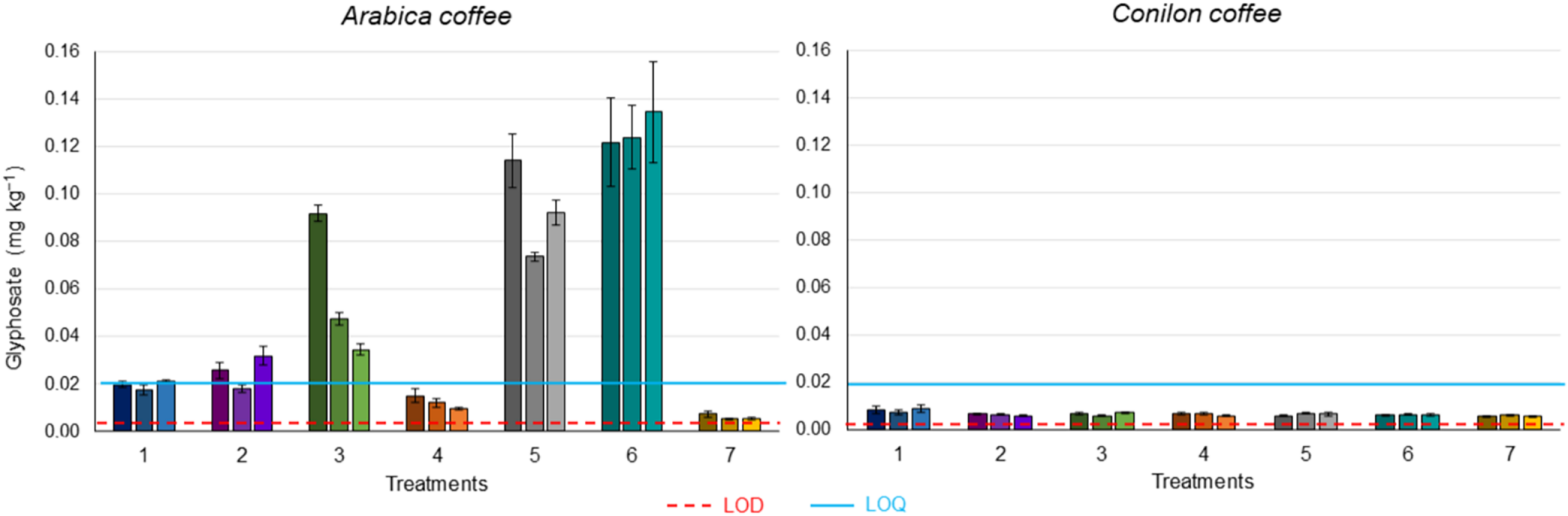
Glyphosate residues found in green Arabica and Conilon coffee beans under different herbicide RoundUp treatments. Each bar indicates a block, with each treatment performed in triplicate, i.e., three blocks. The error bar indicates the variation obtained for authentic triplicates of each block.

Therefore, when the manufacture’s recommendations regarding dosage and preharvest interval are followed, glyphosate residue levels in the green coffee beans tends to be lower than the MRL established by current legislation. However, it is essential to consider the spray drift effects, which may increase active ingredient concentration in the coffee beans, potentially leading to a regulatory noncompliance and posing as a risk to consumers’ health.^7^

The results obtained in this study offer a refined analytical approach for monitoring highly polar herbicides in green coffee, while also contributing to a better understanding of how field application parameters may influence residue levels in different coffee species.

## Supplementary Material



## Notes

Centroid simplex mixture design matrix and its responses; Flowchart of the experiments for evaluating cleanup; Chromatogram obtained for glyphosate, AMPA and glufosinate using the XSelect CSH Fluoro-Phenyl column; Chromatograms obtained for glyphosate, AMPA, and glufosinate under the initial condition and the condition optimized by the Plackett–Burman design (PDF)

## Abbreviations

ACN: acetonitrile
AMPA: aminomethylphosphonic acid
ANOVA: analysis of variance
APP: anionic polar pesticide
DSPE: dispersive solid-phase extraction
ESI: electrospray ionization
EtOAc: ethyl acetate
HILIC: hydrophilic interaction liquid chromatography
HLB: hydrophilic–lipophilic balance
HPP: highly polar pesticide
LC-MS/MS: liquid chromatography-tandem mass spectrometry
LLE: liquid–liquid extraction
LOD: limit of detection
LOQ: limit of quantification
MeOH: methanol
MRL: maximum residue limit
MRM: multiple reaction monitoring
MTBE: methyl *tert*-butyl ether
PVDF: polyvinylidene fluoride
QuPPe-PO: quick polar pesticide-plant origin
RSD: relative standard deviation
SPE: solid-phase extraction
