# Supplementary material for: Simultaneous Determination of Glyphosate, Aminomethylphosphonic Acid, and Glufosinate in Green Coffee Beans by LC-MS/MS: Optimization, Validation, and Field Study
Source: J Agric Food Chem. 2026 Jan 20;74(4):4027–35. doi: 10.1021/acs.jafc.5c09979 (PMC12879917; doi:10.1021/acs.jafc.5c09979)
Supplement: Supplementary file 1 [file jf5c09979_si_001.pdf]

Supporting Information

Simultaneous determination of glyphosate, aminomethylphosphonic acid, and glufosinate in green coffee beans by LC-MS/MS: optimization, validation, and field study

Júlio César R. M. da Silva[1], Millena Christie F. Avelar[2], Márcia C. M. Ribeiro[2], Mariana de O. Almeida[2], Vanessa H. F. de Faria[2], Vanessa M. Osório[3], Adriana F. Faria[1]*

[1] Department of Chemistry – Institute of Exact Sciences – Universidade Federal de Minas Gerais, 31270-901 Belo Horizonte – MG, Brazil.

[2] Pesticides Residues Laboratory – Fundação Ezequiel Dias, 30510-010 Belo Horizonte – MG, Brazil.

[3] Department of Chemistry and Physics – Center of Exact, Natural and Health Sciences – Universidade Federal do Espírito Santo, 29500-000 Alegre – ES, Brazil

* E-mail: adriana@qui.ufmg.br

Content of supporting information

Table S1. Centroid simplex mixture design matrix and its responses (y) 3

Figure S1. Flowchart of the experiments for evaluating cleanup by SPE with C18 (500 mg) and HLB (200 mg), DSPE with C18 (75.00 mg), and their combination with LLE (750 µL of MTBE:EtOAc 9:1, v/v)... 4

Figure S2. Chromatogram obtained for glyphosate (1.0 µg mL^{-1}), AMPA (1.0 µg mL^{-1}) and glufosinate (0.5 µg mL^{-1}) in (a) solvent (MeOH:H_2O 1:1, v/v) and (b) in green coffee extract using the XSelect CSH Fluoro-Phenyl column and the applied gradient: ultrapure water acidified with 0.5% (v/v) formic acid and 0.1% (v/v) InfinityLab Deactivator additive (phase A) and acetonitrile (phase B). 5

Figure S3. Chromatograms obtained for glyphosate (1.0 µg mL^{-1}), AMPA (1.0 µg mL^{-1}), and glufosinate (0.05 µg mL^{-1}) in green coffee extract under the initial condition and the condition optimized by the Plackett–Burman design..... 6

Table S1. Centroid simplex mixture design matrix and its responses (y)

Run	x_1[a]	x_2[b]	x_3[c]	x_{12}	x_{13}	x_{23}	x_{123}	y
1	1	0	0	0	0	0	0	57,171
2	1	0	0	0	0	0	0	56,922
3	1	0	0	0	0	0	0	61,665
4	0	1	0	0	0	0	0	84,842
5	0	1	0	0	0	0	0	86,775
6	0	1	0	0	0	0	0	87,943
7	0	0	1	0	0	0	0	85,479
8	0	0	1	0	0	0	0	82,223
9	0	0	1	0	0	0	0	82,475
10	0.5	0.5	0	0.25	0	0	0	90,288
11	0.5	0.5	0	0.25	0	0	0	90,420
12	0.5	0.5	0	0.25	0	0	0	88,391
13	0.5	0	0.5	0	0.25	0	0	136,866
14	0.5	0	0.5	0	0.25	0	0	133,850
15	0.5	0	0.5	0	0.25	0	0	134,734
16	0	0.5	0.5	0	0	0.25	0	138,241
17	0	0.5	0.5	0	0	0.25	0	147,998
18	0	0.5	0.5	0	0	0.25	0	152,579
19	0.333	0.333	0.333	0.111	0.111	0.111	0.037	100,922
20	0.333	0.333	0.333	0.111	0.111	0.111	0.037	95,102
21	0.333	0.333	0.333	0.111	0.111	0.111	0.037	90,792

The values in the matrix represent the solvent volume fraction (v/v), where 0 corresponds to 0% and 1 to 100%. Main effects for the solvents are defined as: x_1 = hexane, x_2 = EtOAc, x_3 = MTBE.

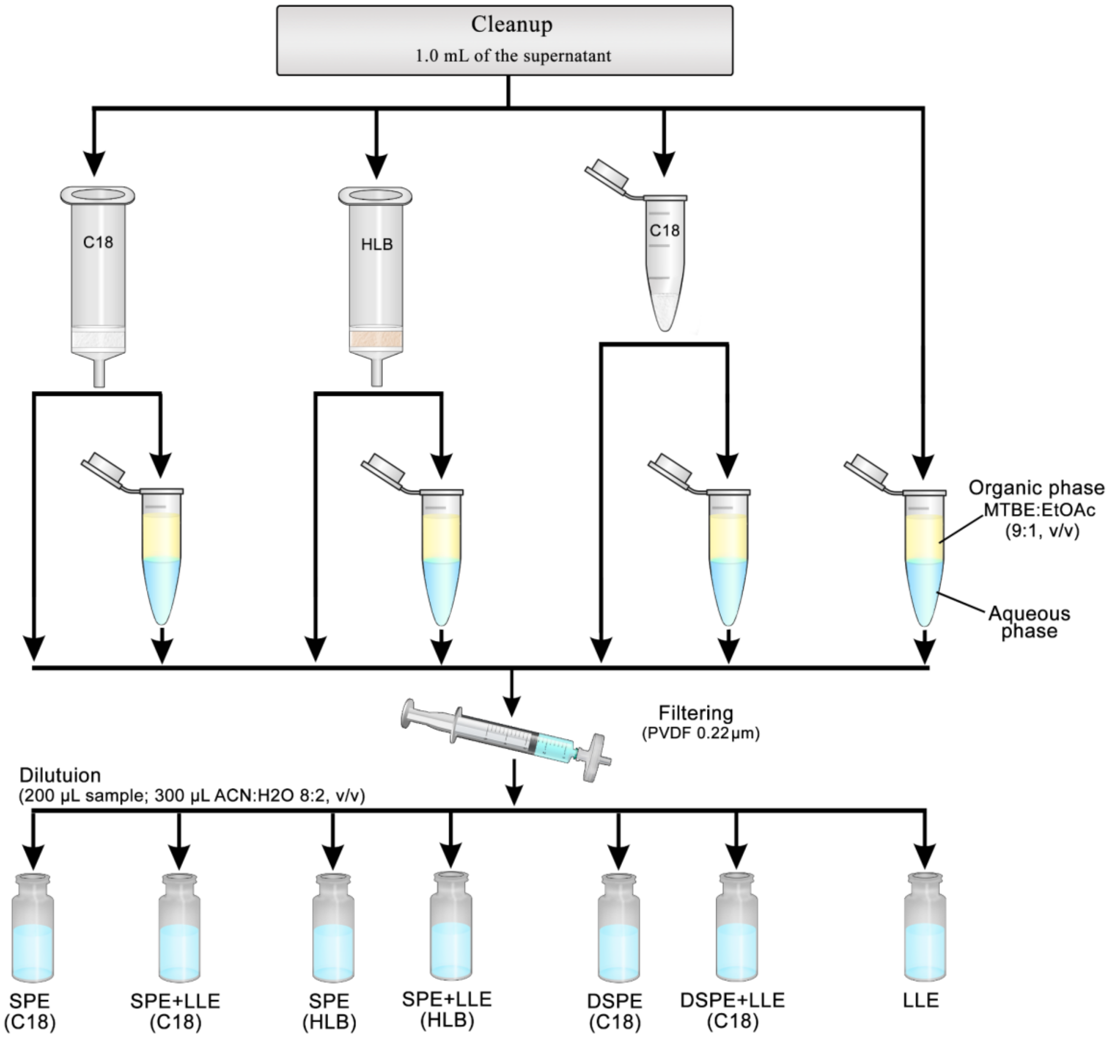

Figure S1. Flowchart of the experiments for evaluating cleanup by SPE with C18 (500 mg) and HLB (200 mg), DSPE with C18 (75.00 mg), and their combination with LLE (750 μL of MTBE:EtOAc 9:1, v/v).

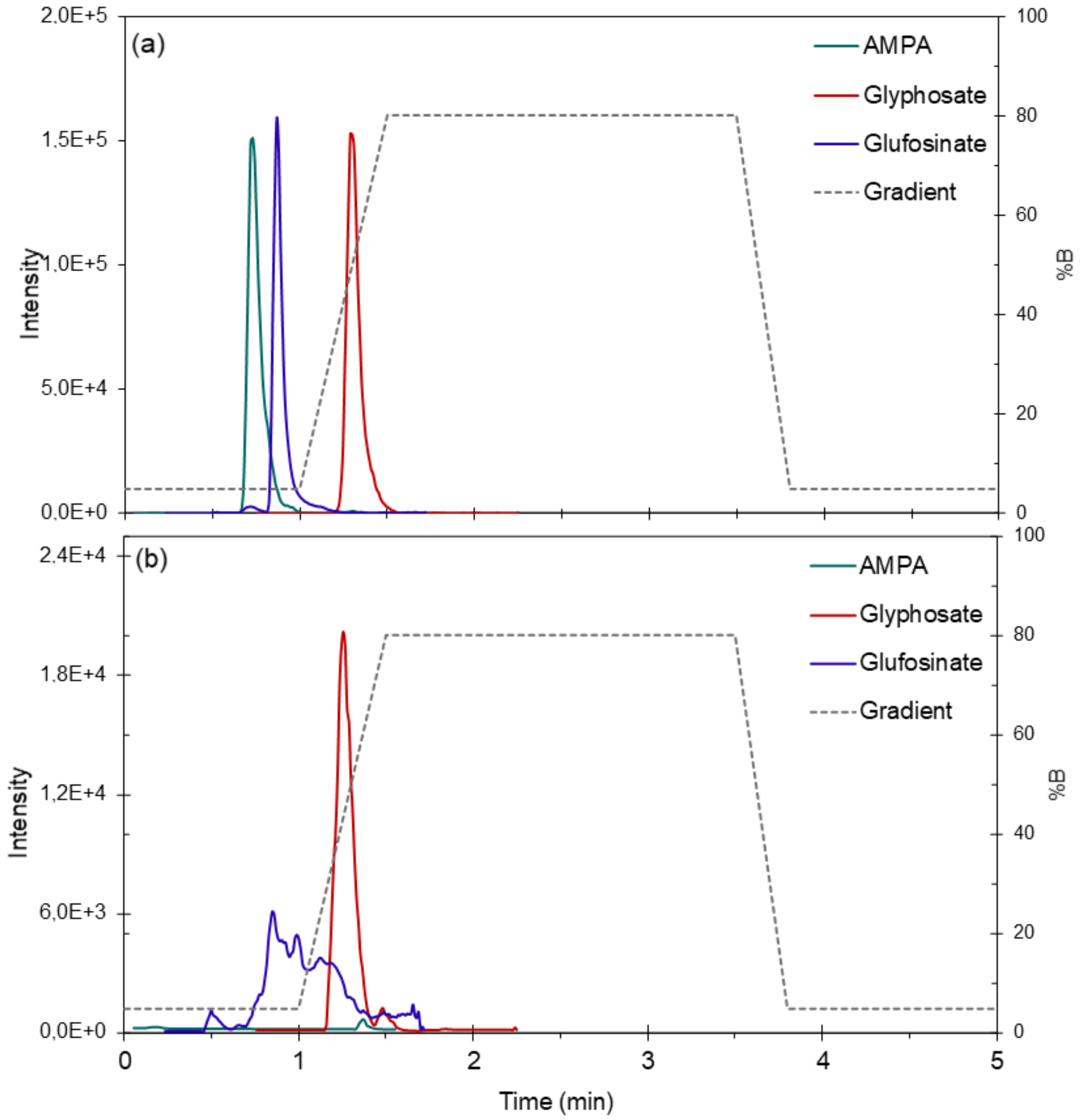

Figure S2. Chromatogram obtained for glyphosate (1.0 µg mL^{-1}), AMPA (1.0 µg mL^{-1}) and glufosinate (0.5 µg mL^{-1}) in (a) solvent (MeOH:H_2O 1:1, v/v) and (b) in green coffee extract using the XSelect CSH Fluoro-Phenyl column and the applied gradient: ultrapure water acidified with 0.5% (v/v) formic acid and 0.1% (v/v) InfinityLab Deactivator additive (phase A) and acetonitrile (phase B).

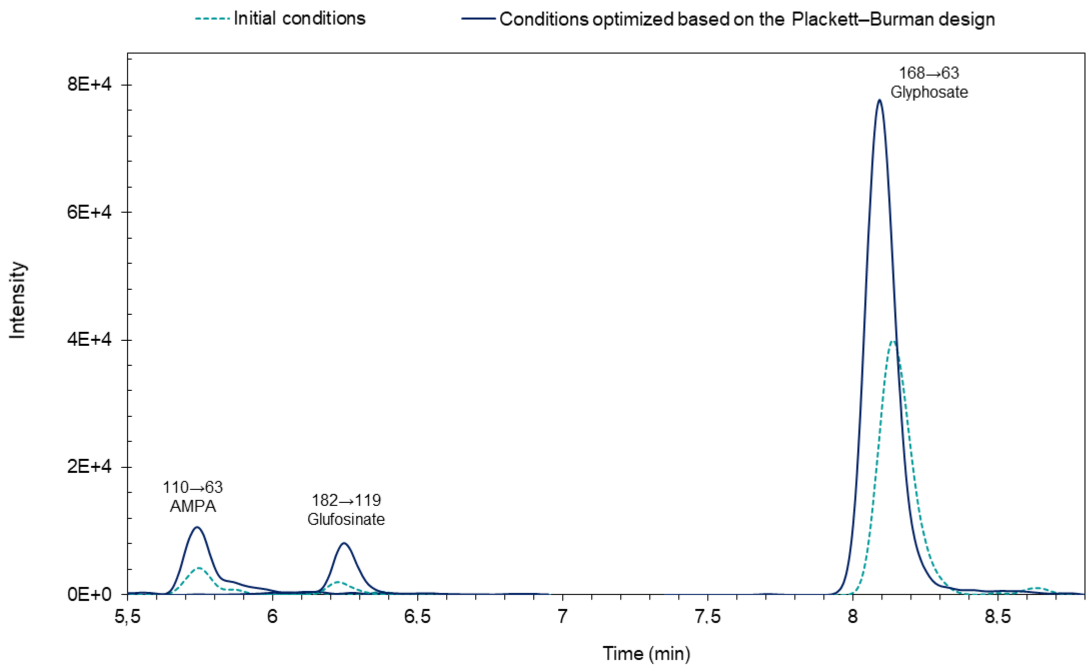

Figure S3. Chromatograms obtained for glyphosate (1.0 µg mL^{-1}), AMPA (1.0 µg mL^{-1}), and glufosinate (0.05 µg mL^{-1}) in green coffee extract under the initial condition and the condition optimized by the Plackett–Burman design.
